# Prior exposure strongly influences mechanisms underpinning survival of heat shock in *Escherichia coli*

**DOI:** 10.3389/fmicb.2025.1644088

**Published:** 2025-10-15

**Authors:** Muhammad Yasir, A. Keith Turner, Sarah Bastkowski, Caroline S. Jarvis, Ryan Sweet, Megan Truong, Ian G. Charles, Mark A. Webber

**Affiliations:** ^1^Quadram Institute Bioscience, Norwich Research Park, Norwich, United Kingdom; ^2^Centre for Microbial Interaction, Norwich, United Kingdom; ^3^Norwich Medical School, University of East Anglia, Norwich Research Park, Norwich, United Kingdom; ^4^Faculty of Science, University of Technology Sydney, Sydney, NSW, Australia; ^5^The University of Sydney, Sydney, NSW, Australia

**Keywords:** heat stress, *Eschericia coli*, TIS, TraDIS-*Xpress*, stepwise heat stress, Tn-seq

## Abstract

The heat shock response of *Escherichia coli* represents a canonical example of how bacteria can recognize a stress and invoke a protective response by altering specific gene regulation. However, most understanding of the processes involved arises from experiments where cells have been subjected to immediate heat shock. In this study, we identified the populations of transposon mutants in *E. coli* BW25113 involved in response to sudden heat shock and stepwise heat stress conditions. We used Transposon-Directed Insertion Site Sequencing with expression (TraDIS-*Xpress*) to identify genes whose function or expression contributed to survival under 5 different heat conditions. These conditions included direct exposure to 44°C, 47°C, or 50° C referred to as “heat shock” or half an hour exposure at 44°C, followed by exposure to 47°C or 50°C referred to as “stepwise heat stress”.A total of 530 genes were identified as contributing to one or more of the heat stress conditions tested, including known heat shock resistance genes. Only 8 genes were common to all 5 conditions, with 4 of these 8 genes being associated with energy generation. The results showed fundamentally different responses between shock and stepwise stress. In heat shock conditions, most genes conferring a fitness benefit contained an increase in insertions (loss of function) as compared to the control (37°C), while in stepwise heat stress, most genes conferring a fitness benefit had fewer insertions (representing protection of function) as compared to the control. Cell envelope genes involved in lipopolysaccharide biosynthesis (*lpxM, lptC*), the Tol-Pal system (*tolABQR-pal*), and outer membrane biogenesis (BAM complex) were detrimental during heat shock but essential for stepwise adaptation, while regulatory genes *relA* (stringent response) and the *rsx* operon (redox regulation) were specifically required for stepwise heat stress response.Prior exposure to sub-lethal heat stress dramatically alters the genetic landscape for survival, allowing energy-intensive adaptive responses rather than the cellular simplification strategies required during immediate heat shock. This work shows that stress responses are dependent on stepwise heat exposure whilst providing significant new information about the sudden heat shock.

## 1 Introduction

*Escherichia coli* serves as a model organism in microbiology and has become central to biotechnology applications like genetic engineering, recombinant protein production, and as a host for cloning vectors (Mandel and Higa, 1970; Sambrook and Russell, 2006). As both a commensal inhabitant of the human gut microbiome and an opportunistic pathogen causing urinary tract infections and foodborne illness, *E. coli* represents a critical intersection between beneficial microbial ecology and public health concerns (Kaper et al., 2004; Tenaillon et al., 2010). Recent advances have further expanded its utility in synthetic biology, metabolic engineering, and as a platform for studying fundamental bacterial physiology (Blattner et al., 1997; Feist et al., 2007).

As with most bacteria, *E. coli* can experience rapid changes in environmental conditions which requires it to be able to mount a variety of stress specific responses which alleviate the impacts of the appropriate environmental challenge (e.g., changes in pH, temperature, and osmotic stress) (Richter et al., 2010). One of the major environmental challenges that *E. coli* experiences is heat stress, which can lead to protein denaturation, altered composition and fluidity of the bacterial membrane, changes in cellular permeability and ion fluxes and sometimes cell death. To survive and adapt to high temperatures, *E. coli* has developed various molecular mechanisms that regulate gene expression, protein folding, and energy metabolism to provide a co-ordinated response to the effects of heat (Weber et al., 2002; Ye et al., 2012).

One of the key responses of *E. coli* to heat is induction of sigma 32 (σ32), which alters the transcriptional programme of the cell and regulates the expression of many genes that encode heat shock proteins (HSPs) and other stress-response (Roncarati and Scarlato, 2017). HSPs act as molecular chaperones that assist in the folding of nascent polypeptides, preventing the aggregation of misfolded proteins and promoting the refolding of damaged proteins. Other mechanisms *E. coli* uses for heat stress tolerance include other chaperones (e.g., DnaK, DnaJ, GrpE), proteases (e.g., ClpXP, HslUV), RNA chaperones (e.g., Hfq), and metabolic enzymes (e.g., pyruvate oxidase) (Guisbert et al., 2008; Roncarati and Scarlato, 2017).

Despite significant progress in understanding the biology of heat shock and heat adaptation in *E. coli*, there are still many unanswered questions, how the timing, rate and duration of the heat shock affect the responses, and how *E. coli* adapts to gradual temperature changes over time as compared to sudden shock. The effects of heat shock can be studied using various techniques, such as survival assays, transcriptomics, proteomics, metabolomics, or microscopy (Haddad et al., 2022; Jozefczuk et al., 2010; Lüders et al., 2009; Riquelme-Barrios et al., 2025). In this study we use TraDIS-*Xpress* to compare fitness of a massive pool of transposon mutants impacting the whole genome simultaneously and compared the landscapes of genes needed to survive heat shock with or without a period of adaptation to an intermediate temperature. This revealed dramatic differences between the two and shows prior exposure has a significant effect on the pathways which are viable for surviving heat shock.

## 2 Materials and methods

### 2.1 Culture and experimental conditions

The *E. coli* strain BW25113 mutant library used in this study was previously described in Yasir et al. (2020). An aliquot of this library stored at −80°C was diluted 1:10 in Luria-Bertani (LB) broth and incubated at 37°C for 20 mins. This sub-culture was further diluted 1:100 in 10 mL of LB broth using 30 mL Universal tubes, with either 0, 0.2, or 1.0 mM of 1M isopropyl β-d-1-thiogalactopyranoside (IPTG). Each condition was prepared in duplicates and incubated overnight at 37°C, 44°C, 47°C, or 50°C, shaking at 200 rpm for heat shock experiment and for gradient heat, incubated at 44°C for half an hour and then incubated at 47°C or 50°C (Supplementary Figure 1).

The temperature range of 37°C (control), 44°C, 47°C, and 50°C was selected based on preliminary colony counting experiments across 37–55°C that revealed mutant survival drops drastically above 45°C, and previous work by Murata et al. (2011) demonstrating that Keio collection mutants struggle to grow at 45°C but die at 46°C, establishing our range as optimal for rigorous stress analysis.

To determine cell numbers for each condition, cultures were serially diluted 10-fold to a dilution of 10^−7^ in a 96-well plate. Five microliter volumes were spotted onto LB agar and incubated at 37°C overnight. For DNA extraction and sequencing, cultures were centrifuged in 2 mL deep 96-well plates at 4,500 rpm for 20 mins, and stored at −20°C.

### 2.2 DNA extraction and sequencing

After growth, DNA was extracted from each experiment using a Quick-DNA™ Fungal/Bacterial 96 Kit (Zymo Research). Genomic DNA from the transposon mutant library at different temperature conditions was diluted to 11.1 ng/μL and tagmented using MuSeek DNA fragment library preparation kit (ThermoFisher, USA). Fragmented DNA was purified using AMPure XP (Beckman Coulter, USA). DNA was amplified by PCR using biotinylated primers specific to the transposon and primers for the tagmented ends of DNA. PCR products were purified again using AMPure XP beads and incubated for 4 h with streptavidin beads (Dynabeads) to allow for capture of the DNA fragments with the transposon. A subsequent indexing PCR step using barcoded sequencing primers allowed for the pooling of samples. Streptavidin beads were magnetically removed from the PCR products which were further purified and size-selected using AMPure XP beads. The indexed library was quantified using Qubit 3.0 (Invitrogen, USA) and Tapestation (Agilent Technologies, USA). The library was sequenced using NextSeq 500 Illumina machine with a NextSeq 500/550 High Output Kit v2.5 (75 cycles) (Illumina).

### 2.3 Data processing and analysis

**Figure 1:**
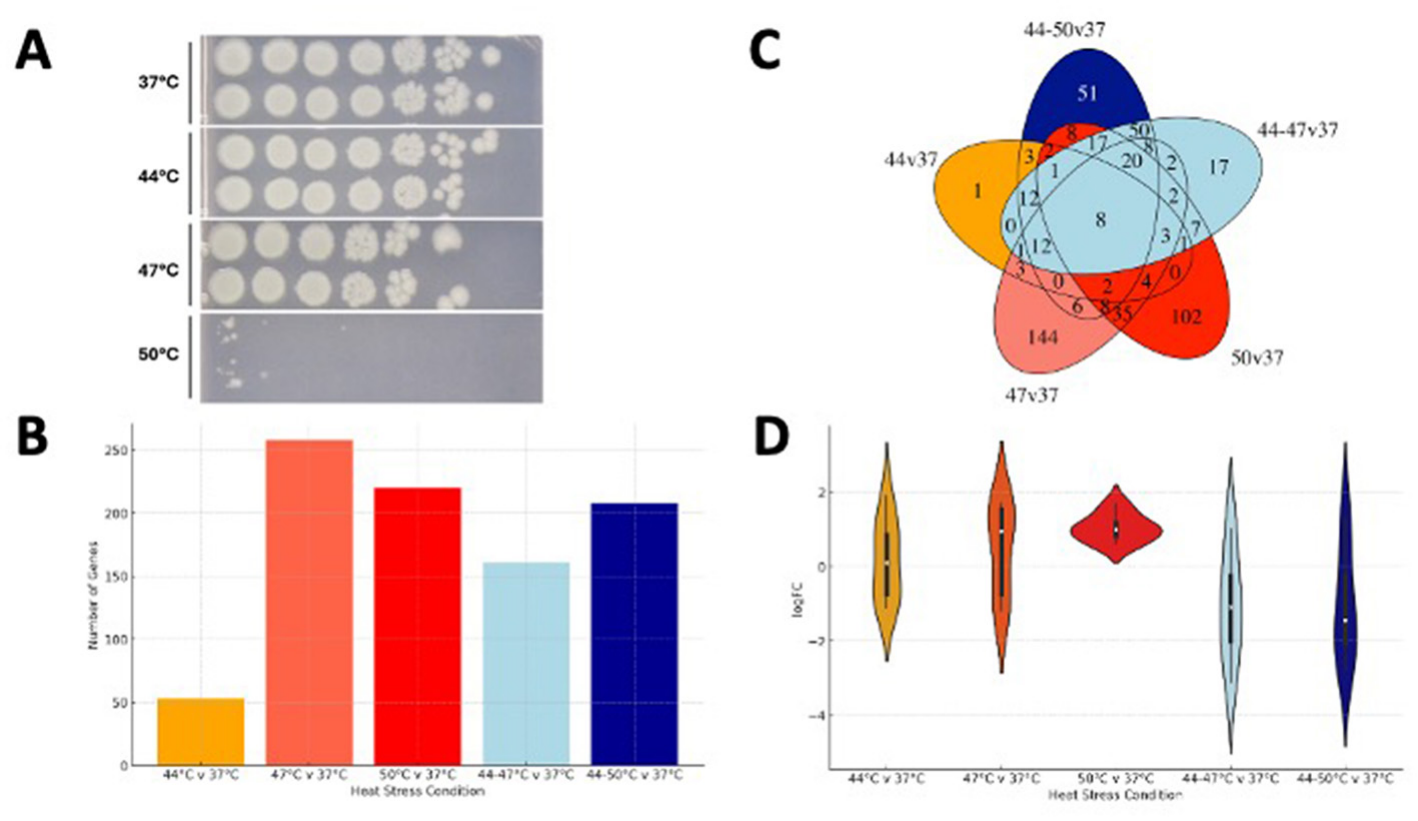
Transposon library survival under different heat stress conditions. **(A)** shows numbers of viable cells of *E. coli* BW25113 in each stress condition (5 μl spots inoculated from a 10-fold dilution series on LB agar). **(B)** The number of genes identified as significantly important in each stress condition. **(C)** A Venn diagram of all stress conditions indicating overlapping genes. **(D)** The log FC in transposon mutant abundance within the 8 genes present in all five stress conditions.

**Figure 2:**
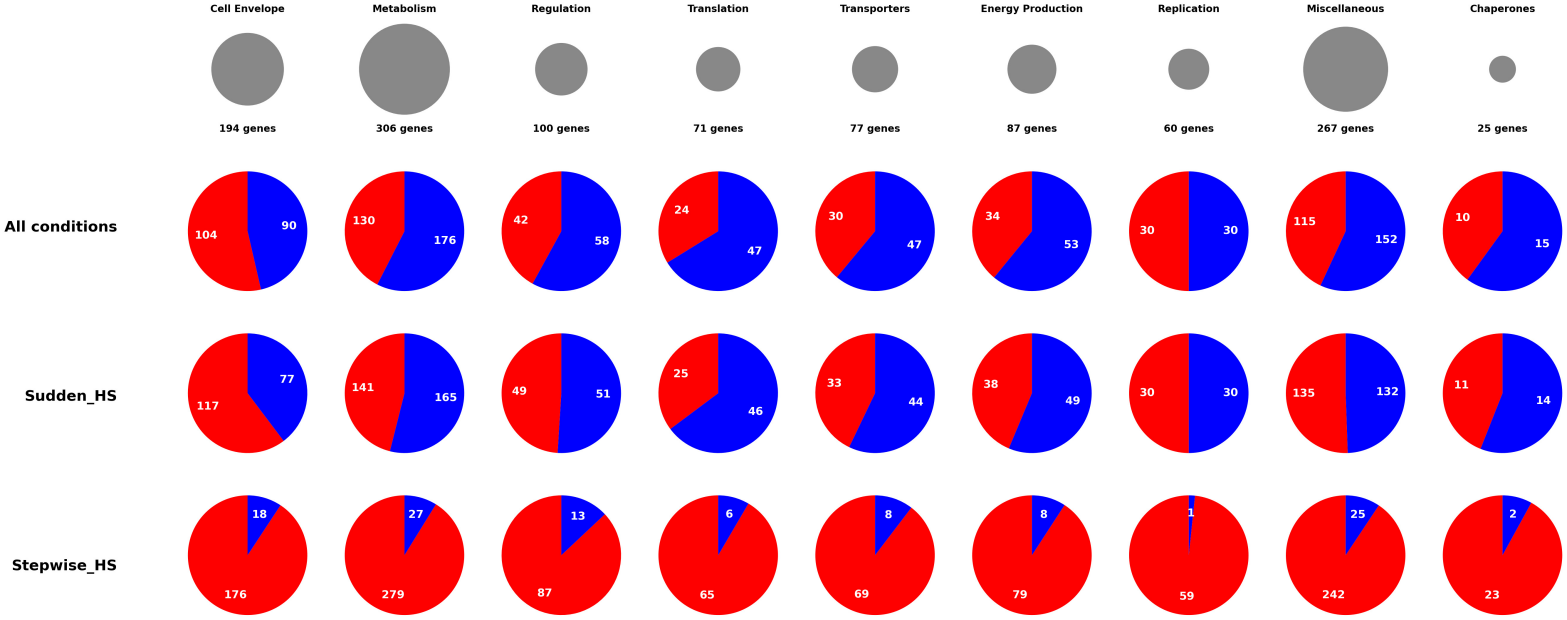
Pie chart showing the number of genes important under heat stress condition. In the top row, gray circles show the total number of genes of different categories important under any stress condition. The pie charts represent the fraction of essential (red) and non-essential (blue) genes in sudden heat shock or stepwise heat stress conditions in each of the functional categories. The numbers represent the genes in each category of the pie charts. The size of the circles in the top row indicates the number of mutants important in survival in each category.

**Figure 3:**
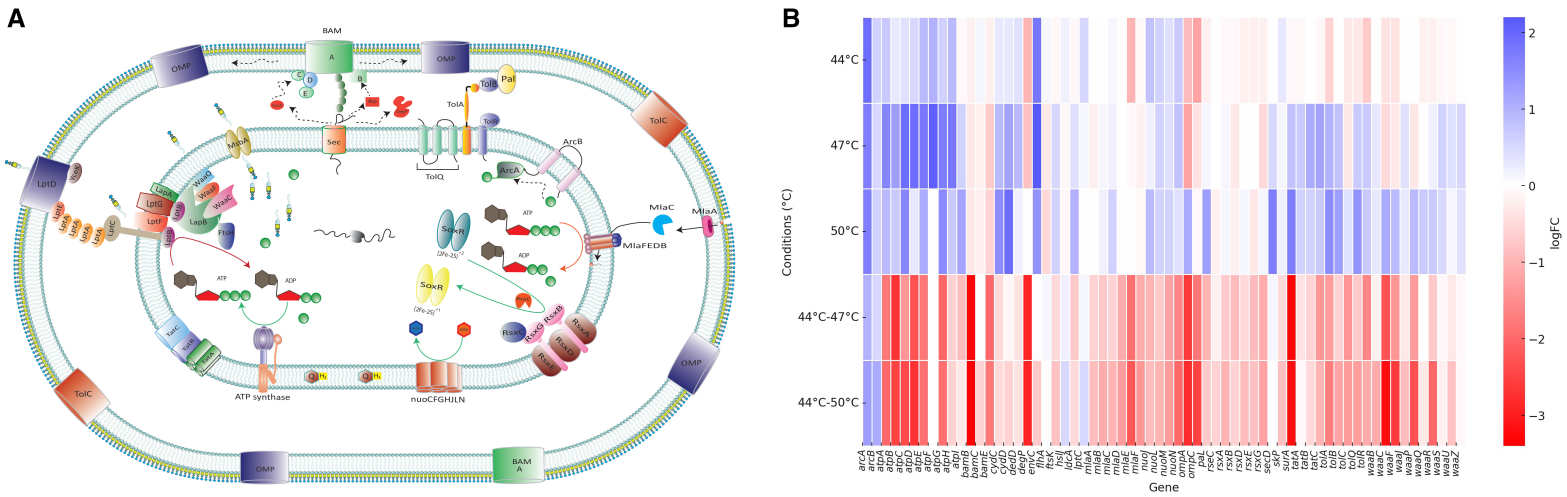
Cell envelope response to heat stress conditions. **(A)** Schematic representation of the *E. coli* cell envelope showing proteins identified as important for heat stress response. Genes that are preserved or disrupted under different temperature conditions are detailed in **(B)** Arrows within the cytoplasmic membrane represent the direction of biochemical reactions, while arrows in the periplasmic space indicate the direction of molecular transport. **(B)** Heat map showing log fold change (logFC) of transposon insertions (relative to control) in genes encoding cell envelope proteins depicted in panel **(A)** Positive logFC values (blue) indicate genes that were disrupted/mutated, while negative logFC values (red) indicate genes that were preserved/protected under heat stress conditions. Enzyme encoded by each gene abbreviated in the heat map or the functions of the locus can be found in Supplementary Table 5, column B.

Sequencing data were analyzed using the established TraDIS-*Xpress* pipeline with BioTraDIS (version 1.4.1) and AlbaTraDIS (version 0.0.5) as described in Page et al. (2020). BioTraDIS aligned sequence reads against the reference genome (CP009273) using the BWA aligner and created insertion plots of mapped transposon insertion sites. AlbaTraDIS compared the number of inserts within each gene between conditions and controls to generate log_2_FC and q-values. Normalization and statistical testing were performed as part of the AlbaTraDIS pipeline. The AlbaTraDIS output files provide a preliminary annotation sourced from the reference genome in EMBL format in the pipeline (Page et al., 2020). This was supplemented with information from ecocyc.org when additional interpretation was required. Genes were filtered using log2FC ± 0.5 and q-value < 0.05. Data visualization for Figures 1–3 was performed using standard plotting scripts (https://github.com/yaasircheema/Scripts_for_TraDIS_HS_data_visualization). Insertion patterns at candidate loci were visually inspected using Artemis (Carver et al., 2012).

## 3 Results

### 3.1 Heat stress at different temperatures

Survival of a panel of *E. coli* BW25113 transposon mutants was compared at a range of temperatures; 37°C, 44°C, 47°C, and 50°C (sudden heat shock) after overnight incubation in LB broth. In addition, a series of samples were incubated for 30 min at 44°C before then being exposed to 47°C or 50°C (Supplementary Figure S1) allowing us to compare the response to “heat shock” with “stepwise heat stress”. All samples were compared to 37°C as a reference. Samples were plated out on LB agar to assess survival by colony counting and mutant abundance enumerated from DNA preparations. The colony counts showed no difference in mutants survival between 37°C and 44°C before survival started decreasing at 47°C with a marked reduction in viable cells at 50°C (Figure 1A).

### 3.2 Many genes were identified as relevant to heat stress

A total of 530 genes were identified as being relevant to either heat shock or stepwise heat stress based on a significantly different abundance of mutants within the transposon mutant libraries after exposure, compared to the 37°C control. The highest numbers of mutants identified were after exposure to a 47°C shock and the smallest number were identified after shock at 44°C (Figure 1B, Supplementary Table 1). The Venn diagram illustrates results from all the stress conditions shows only 8 genes were common to all 5 conditions (Figure 1C, Supplementary Table 2). Four of these (*arcA, sucA, atpA* and *gltA*) are involved in energy metabolism, *dnaK* is a gene that encodes chaperone involved in protein folding, *ompA* encodes a major outer membrane protein, *qseC* is part of a two-component system and *xerC* plays a role in cell division (Figure 1D, Supplementary Table 3).

### 3.3 Shock and stepwise heat stresses select very different sets of genes

The 530 genes identified as playing a significant role in one or more of the conditions were grouped according to their functional annotation (Figure 2, Supplementary Table 4). This resulted in 9 major groups. Within these we analyzed the number of genes with either more or less transposon insertions. Those where an increase in mutants provides better growth as compared to control are referred as non-essential (shown as blue in pie charts) and those where the function of the gene was protected as compared to the control are referred to as essential for heat stress (shown as red in pie charts). In Figure 2, the top panel shows the different groups of genes with different sizes of the circles corresponding to the number of genes in each category. Genes involved in metabolism represent the largest category followed by genes involved in the cell envelope. The smallest group was the chaperone genes. In heat shock conditions, each group shows that most of the significant genes were non-essential. Markedly, there was a major difference in the patterns of inserts between shock and stepwise stresses in all categories. Most functions were predicted to be detrimental (non-essential) in heat shock whilst under stepwise heat stress, most significant genes were essential.

### 3.4 The cell envelope has opposite roles in shock and stepwise heat stress

The shift in *E. coli* behavior under heat shock as compared to stepwise heat stress could be explained by significant metabolic and structural changes between the two. *E. coli* mutants lacking major envelope functions survived significantly better in heat shock. This included mutants lacking functional genes involved in outer membrane biogenesis and integrity, lipopolysaccharide (LPS) synthesis (including glycosyltransferases; lipid A acylation and transportation components); the maintenance of lipid asymmetry (MLA) system; the twin-arginine translocation pathway (*tatABC*); SecD (part of the Sec translocon), and the Tol-Pal system (*tolABQR-Pal*) (Figure 3). Similarly, the loss of gene functions encoding the outer membrane biogenesis machinery, including the genes encoding β-barrel assembly machine (BAM) complex, chaperones responsible for folding proteins in the periplasmic space, and outer membrane proteins (OMP) was beneficial under heat shock.

*E. coli* also survived better under heat shock by mutations in the genes encoding certain proteins embedded in the inner membrane, for example the electron transport chain components ATP synthase; cytochrome oxidase; NADH oxidoreductase and cell replication machinery proteins involved in septal ring formation; including the ABC transporter components (FtsX and FtsE); DNA translocase (FtsK); transglycosylase (FtsW); the division protein (FtsN), and Min system (MinCDE), which prevents septal ring formation at the cell poles.

In marked contrast, in stepwise heat stress *E. coli* many of the genes encoding the same systems were protected, indicating their importance for survival after the period of adaptation at 44°C (Figure 3B). This included genes responsible for lipopolysaccharide (LPS) synthesis; lipid A acylation (e.g., *lpxM*), and transportation (e.g., *lptC*). The maintenance of lipid asymmetry (MLA) system; twin-arginine translocation pathway (*tatABC*); and SecD; the Tol-Pal system (TolABQR-Pal) and other structural proteins encoding genes including *slp, slyB*, and *lpp*, which contribute to outer membrane stability (Figure 3A). The genes encoding the outer membrane biogenesis machinery, including the β-barrel assembly machine (BAM) complex, chaperones responsible for folding proteins in the periplasmic space, and outer membrane proteins were also protected during stepwise heat stress (Figure 3B).

The genes encoding electron transport chain components ATP synthase, cytochrome oxidase, NADH oxidoreductase and fumarate reductase became essential for stepwise heat stress. This we interpret to indicate the cell can mount an active response to the stress after being exposed to 44°C and that this required significant energy and metabolic activity. For example, the redox system (Rsx complex) is embedded in the inner membrane and plays a key role with RseC in the regulation of SoxR, a redox-sensitive transcription factor that regulates the transcription of *soxS* and became important after heat adaptation.

Moreover, in stepwise heat stress, genes encoding various parts of the cellular replication machinery were protected including proteins involved in septal ring formation, such as the ABC transporter components (FtsX and FtsE), the periplasmic chaperone (FtsP), DNA translocase (FtsK), transglycosylase (FtsW), the division protein (FtsN) and the Min system (MinCDE), which prevents septal ring formation at the cell poles helps cell to proliferate better. This suggests that the adaptation to 44°C allows cells to mount a response which requires energy generation but also enables continued growth rather than just survival.

Together, the contrast between the two types of stress illustrates a divergence in viable survival strategies between heat shock and stepwise adaptation. The data suggest options to survive the more severe shock are limited with the cell envelope being a major constraint and suggest major modifications of the cell envelope are required to survive heat shock. This data is consistent with one of the routes to surviving heat shock being to lose the cell wall whilst in stepwise heat stress, the period of adaptation allows induction of a protective set of responses which allow *E. coli* to proliferate normally.

### 3.5 Metabolism and gene regulation were also involved in heat stress adaptation

*E. coli* mutants survived better under heat shock when there were mutations in the genes encoding amino acid synthesis pathways including the methionine synthesis pathway (methionine synthase [MetE] and 5, 10-methylenetetrahydrofolate reductase [MetF], the transcriptional repressor [MetJ], and homoserine O-succinyltransferase [MetL]). Similarly, disruption of aromatic amino acids biosynthesis (phenylalanine, tyrosine, and tryptophan), such as 3-phosphoshikimate 1-carboxyvinyltransferase (AroA), 3-dehydroquinate dehydratase (AroD), chorismate synthase (AroC), 3-deoxy-D-arabino-heptulosonate-7-phosphate synthase (AroH) Glutamine synthetase (GlnA) and other amino acids such as Aspartate ammonia-lyase (AspA) and aspartate aminotransferase (AspC) helped *E. coli* survive heat shock better (Supplementary Table 5).

Disruption of genes encoding carbohydrate metabolism, malate dehydrogenase (Mdh) and citrate synthase (GltA) in heat shock were helping *E. coli* survive under heat shock better. In contrast, the genes encoding fumarase C (FumC), 2-oxoglutarate dehydrogenase (SucA), dihydrolipoyl transsuccinylase (SucB), and succinyl-CoA synthetase (SucC) that are involve in succinate and fumarate metabolism were protected during sudden heat shock (Supplementary Table 5).

A series of other biosynthesis and transportation systems that are metabolically expensive for *E. coli* were inactivated during heat shock by mutation in the genes encoding the GMP synthase (GuaA), IMP dehydrogenase (GuaB), permease (PstA), ATP-binding proteins (PstB and PstC), and the phosphate-binding protein (PstS). These amino acid and metabolic pathways were all protected in the stepwise heat stress.

## 4 Discussion

Current study finds fundamental differences in the genetic requirements for *E. coli* BW25113 survival under immediate heat shock vs stepwise heat stress, providing the first genome-wide comparison of these distinct stress response strategies. Our TraDIS-*Xpress* analysis identified 530 genes contributing to heat stress survival across five temperature conditions, with only eight genes being universally important (Supplementary Table 3). We also discovered the genetic landscape for heat stress survival is dramatically altered by prior exposure to sub-lethal temperatures.

The core finding of this work deduced from genetic mutations is the opposite roles of cellular systems depending on adaptation history. Under immediate heat shock, survival was enhanced by loss-of-function mutations in genes encoding cell envelope components, energy generation systems, replication and biosynthetic pathways, suggesting a survival strategy that prioritizes cellular simplification and persistence over normal growth. Conversely, stepwise heat adaptation required protection of these same functions, enabling cells to maintain normal cellular architecture and proliferation at high temperature stresses. This dichotomy indicates that *E. coli* employs fundamentally different survival strategies under a “crisis mode” response during heat shock vs a “regulated adaptation” response when given time to prepare for the shock at the same temperature (Figure 2).

Our data strongly suggest that immediate heat shock survival may involve defective cell envelope (L-form-like mechanisms), where cells survive by losing cell wall integrity and conventional division machinery. This interpretation is supported by the beneficial loss of Tol-Pal system components, BAM complex constituents, LPS biosynthesis genes, and cell division apparatus all consistent with cell wall-deficient survival states. This represents a previously under reported extreme adaptation mechanism in *E. coli* heat stress responses. Moreover, the loss of function of replication machinery indicates the surviving and thriving might not be a good idea under “sudden crisis” and cells go in persistence mode.

The identification of regulatory circuits governing the transition between shock and stepwise responses particularly the essential roles of *relA* and the *rsx* operon in stepwise adaptation provides molecular targets for future investigations into stress response switching mechanisms. These regulators appear to be central in the cell ability to mount energy intensive protective responses rather than resorting to cellular simplification as it happened in sudden heat shock.

Our findings complement and extend classical heat shock research while revealing previously unappreciated complexity. The established σ32-mediated heat shock response, characterized by upregulation of chaperones like DnaK, GroEL, and proteases (Guisbert et al., 2008; Roncarati and Scarlato, 2017), represents the well-studied adaptive pathway that we now understand requires prior conditioning to be effective. Our observation that DnaK is among the eight universally important genes validates this classical knowledge while placing it in a broader context.

The protective role of the electron transport chain and TCA cycle components in stepwise adaptation aligns with previous metabolomic studies showing that energy metabolism reprogramming is crucial for temperature adaptation (Weber et al., 2002; Ye et al., 2012). However, our demonstration that these same systems become detrimental during immediate shock reveals why previous studies focusing on gradual temperature increases may have missed the crisis response mechanisms we identified.

Our L-form survival hypothesis is supported by previous observations of membrane alterations during heat stress (Markova et al., 2010; Tonyali et al., 2019; Yuk and Marshall, 2003), but extends these findings by suggesting that controlled cell wall loss may be an active survival strategy rather than simply stress-induced damage. This connects to emerging research on bacterial cell wall plasticity and alternative survival states.

The regulatory insights regarding *relA* and the stringent response align with growing evidence that (p)ppGpp-mediated signaling is crucial for coordinating stress responses (Irving et al., 2021). Our finding that this system is dispensable during shock but essential for adaptation suggests that stringent response activation requires cellular energy reserves that immediate shock conditions do not permit.

Our TraDIS-*Xpress* approach provides complementary insights to traditional omics methods used in heat stress research. While transcriptomic studies reveal gene expression changes during heat shock (Jozefczuk et al., 2010; Riquelme-Barrios et al., 2025), they cannot distinguish between genes that are upregulated as part of a protective response vs those that are upregulated but functionally irrelevant to survival. Our fitness-based approach directly identifies genes whose function is critical for survival, regardless of their expression patterns. Similarly, proteomic approaches (Lüders et al., 2009) can identify which proteins accumulate during heat stress but are limited by detection sensitivity and cannot easily distinguish protective from detrimental protein changes. Metabolomic studies (Weber et al., 2002; Ye et al., 2012) reveal the downstream consequences of heat stress on cellular metabolism, but our approach identifies the genetic determinants that drive these metabolic changes. However, TraDIS-*Xpress* cannot provide the mechanistic details of gene regulation, protein interactions, or metabolic flux changes that these other approaches reveal. The integration of our fitness-based genetic screen with transcriptomic, proteomic, and metabolomic data would provide the most comprehensive understanding of heat stress adaptation mechanisms.

Our findings highlight the profound influence of prior exposure on bacterial stress adaptation and reveal how this alters the landscape of possible responses. Additionally, we suggest based on a genome wide screen, the critical role of multiple pathways involved in cell envelope production as an extreme adaptive mechanism for surviving heat shock. These findings may be applied to *E. coli* strains used in biotechnology and food safety as these provide a wider understanding than conventional heat stress response.

## 5 Limitations

Although TraDIS-*Xpress* is a robust method for genome wide analysis, this study is limited by analysis of a single laboratory strain (BW25113), which may not represent the diversity of heat stress responses across *E. coli* strains, particularly pathogenic or environmental isolates that may have evolved different survival strategies. Additionally, the proposed L-form survival mechanism during heat shock remains speculative, as direct morphological evidence of cell wall alterations and complementary biochemical validation were not conducted in this study.

## Data Availability

Nucleotide sequence data supporting the analysis in this study has been deposited in ArrayExpress under the accession number E-MTAB-15219. The authors confirm all supporting data, code and protocols have been provided within the article or through supplementary data files. Data visualization scripts can be accessed on “https://github.com/yaasircheema/Scripts_for_TraDIS_HS_data_visualisation”.
