# Supplementary material for: Prior exposure strongly influences mechanisms underpinning survival of heat shock in Escherichia coli
Source: Front Microbiol. 2025 Oct 15;16:1644088. doi: 10.3389/fmicb.2025.1644088 (PMC12568643; doi:10.3389/fmicb.2025.1644088)
Supplement: Supplementary file 1 [file Data_Sheet_1.zip › Datasheet 1/Suppl_Figure1.docx]

**Supplementary figures**


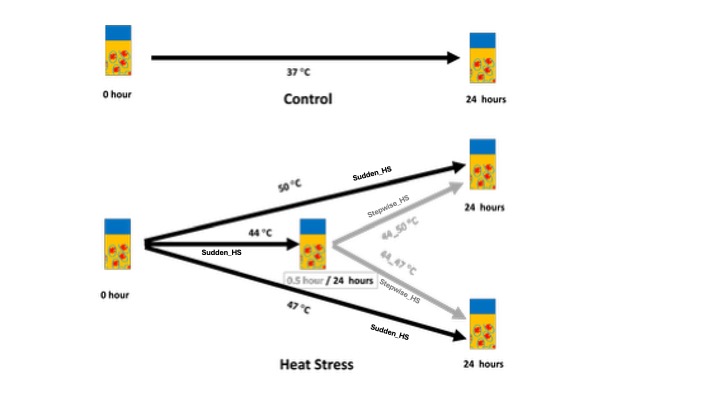


Figure S1. **Experimental setup for heat stress experiment**

This figure illustrates the experimental setup used to subject E. coli cultures to heat stress conditions. Top panel represents control where the library was incubated at 37°C for 24 hours. For test, the library was exposed to 44°C, 47°C and 50°C for heat shock conditions, indicated by black arrows and labelled as Sudden_HS (Sudden heat shock) and separate library aliquots were subject to an initial heat stress of 44°C for 0.5 hours before exposure to higher temperatures of 47°C and 50°C to assess the survival and adaptation of bacteria at varying degrees of heat stress, indicated by grey arrows and labelled with Stepwise_HS (Stepwise heat stress).
